# Material sensitivity of patient-specific finite element models in the brace treatment of scoliosis

**DOI:** 10.3389/fbioe.2023.1111449

**Published:** 2023-02-16

**Authors:** Wenqing Wei, Tianyuan Zhang, Junlin Yang, Yu Qian, Yating Dong

**Affiliations:** ^1^ School of Health Science and Engineering, University of Shanghai for Science and Technology, Shanghai, China; ^2^ Spine Surgery Center, Xinhua Hospital, Shanghai Jiao Tong University School of Medicine, Shanghai, China

**Keywords:** scoliosis, finite element analysis, spine, brace, biomechancis

## Abstract

**Objectives:** To study the mechanical sensitivity of different intervertebral disc and bone material parameters and ligaments under different force configurations and magnitudes in the scoliosis model.

**Methods:** The finite element model of a 21-year-old female is built using computed tomography. Local range of motion testing and global bending simulations are performed for the model verification. Subsequently, Five force of different directions and configurations were applied to the finite element model applying the brace pad position. The material parameters of the model were related to different spinal flexibilities and included different material parameters of cortical bone, cancellous bone, nucleus and annulus. The virtual X-ray technique measured Cobb angle, thoracic Lordosis, and lumbar Kyphosis.

**Results:** The difference in peak displacement is 9.28 mm, 19.99 mm, 27.06 mm, 43.99 mm, and 50.1 mm under five force configurations. The maximum Cobb angle difference due to material parameters are 4.7° and 6.2°, which are converted to thoracic and lumbar in-brace correction difference of 18% and 15.5%. The maximum difference in Kyphosis and Lordosis angle is 4.4° and 5.8°. The average thoracic and lumbar Cobb angle variation difference in intervertebral disc control group is larger than that in bone control group, while the average Kyphosis and Lordosis angle is inverse. The displacement distribution of models with or without ligaments is similar, with a peak displacement difference of 1.3 mm in C5. The peak stress occurred at the junction of the cortical bone and ribs.

**Conclusion:** Spinal flexibility largely influences the treatment effect of the brace. The intervertebral disc has a greater effect on the Cobb angle, the bone has a greater effect on the Kyphosis and Lordosis angles, and the rotation is affected by both. Patient-specific material is the key to increasing accuracy in the personalized finite element model. This study provides a scientific basis for using controllable brace treatment for scoliosis.

## 1 Introduction

Scoliosis is a musculoskeletal disorder characterized as a three-dimensional (3D) deformity with a prevalence of 0.47%–5.2% in adolescents, and a higher incidence in females (Konieczny et al., 2013; Wei et al., 2022). Although many potential etiologies of scoliosis have been proposed, including biomechanical, genetic, and neonatological; the primary etiology is unknown (Fadzan and Bettany-Saltikov, 2017). Curve progression deforms the patient’s back, and depending on the severity, may lead to psychological problems, back pain, local motor dysfunction, and cardiovascular disease (Aebi, 2005; Colak et al., 2017). The Scoliosis Research Society (SRS) categorizes AIS treatment into observation, conservative methods, and surgery (Negrini et al., 2015; Kaelin, 2020).

If the curve ranges from 20° to 45°, bracing is the only acknowledged non-surgical treatment that decreases the probability and affects the coronal curvature of scoliosis patients (Karavidas, 2019). In addition to the casting method, the development of computer-aided design and manufacturing (CAD-CAM) and CAD and finite element modeling (CAD-FEM) has increased the number of options for brace manufacture (Bidari et al., 2021). According to previous studies, the Cobb angle is better corrected by CAD-FEM and CAD-CAM methods compared with conventional methods (Weiss and Kleban, 2015). Although the braces designed using CAD-FEM have been put into clinical use, long-term effects have not been comprehensively evaluated. Thus far, only Guy et al. (2021) found clinical outcomes to be similar to or better than previously reported brace-wearing studies in patients treated with CAD-CAM-designed stents (with or without FEM) using a Randomized Controlled Trial (RCT) in 2 years (*n* = 94 brace-wearing patients). The In-Brace correction (IBC), which partly determines long-term outcome, is related to the experience of the designer. Using FEM to predict IBC not only directly improves the brace treatment, but also allows the clinician to participate in the brace design to further explore the relationship between brace design and long-term outcome.

The SRS generally does not recommend bracing for adult patients with scoliosis. However, some progress has been made in brace treatment for adults. Weiss et al. (2016) published a case report on bracing and exercise treatment for a 37-year-old female patient. In a 16-month follow-up, the Cobb angle of the lumbar Kyphosis improved from 50° to 32°. Papadopoulos (2013) found that in 53% (*n* = 144 adults) of the patients, the Cobb angle improved from 9% to 23%. Mauroy et al. (2016) showed that 24% (*n* = 158 adults) of patients with scoliosis improved more than 5° after 5 years of brace treatment. Thus, Non-growth factors of orthosis can be obtained through adult cases.

The finite element method (FEM) is a numerical simulation method that was first used in the treatment of spinal biomechanics in 1972 (Cui et al., 2022). Since the representative comprehensive volumetric models, including the entire spine and rib, are time-consuming in 3D reconstruction and calculation; studies based on representative segmental volumetric models, typically 1 motion segment, are more commonplace in spinal biomechanics (Wang et al., 2014). Because of the structural complexity of scoliosis, segment modeling cannot adapt to the curve change of the whole spine; therefore, comprehensive models are required in brace treatment. Clin et al. (2011) first applied gravitational loads to show the importance of biomechanical brace action in preventing the scoliotic spine from bending under gravitational forces. A comprehensive personalized FEM, including the heart, liver, spleen, and other organs was built by Guan et al. (2020). This provided the scientific basis for designing a new corrective brace for scoliosis treatment. Karimi et al. (2019) applied various force configurations on the spinal curves, which may allow for automated orthotic selection in future studies. The biomechanical behaviour of a scoliotic spine may not be the same and sensitivity analysis is required to improve the accuracy of the finite element model.

The CAD-FEM brace can be designed to be more controllable to achieve a better treatment effect, thus one of the main issue to meet precision medicine is to assess the impact of the patient’s own flexibility. This study is a material sensitivity analysis based on FEM for a scoliosis patient. Due to the different range of spinal flexibility among patients, it is difficult to obtain an accurate prediction of results in conventional finite element analysis. The spinal flexibility in terms of material distribution is related to the material parameters of the bone, disc and ligament of the spine. Therefore, to improve the accuracy of brace treatment, setting different material parameters by FEM suggest a better understanding on the relationship between spinal material flexibility and clinical indicators in brace treatment. This method highlights the importance of patient-specific materials in accurate finite element models and provides a theoretical basis for designing more controllable bracing.

## 2 Materials and methods

### 2.1 Establishment of the finite element model for scoliosis

The patient was a 21-year-old female with scoliosis, with a thoracic Cobb angle of 26° and a lumbar Cobb angle of 40° measured by X-ray images. The computed tomography (CT) scanning was approved by the relevant ethics board (Approval No. XHEC-D-2021-150). GE Revolution CT was used to scan the patient (0.625 mm slice thickness) from C1 to the pelvis. Elementary point cloud models of each bone structure were extracted according to the thresholding Hounsfield unit of bone. Here, the separated bone was connected using region growing processes, wrapping, and smoothing in medical image processing software (Mimics 19.0; Materialise, Leuven). Reverse engineering software (Geomagic Wrap 2021; Geomagic, America) converted whole vertebral bodies to a 3D geometric model for the established intervertebral disc and set cortical bone thickness to 0.8 mm. In computer-aided design software (Solidworks 2021; Dassault, America), according to the anatomical structure and location of the upper and lower surfaces of the vertebral body, the intervertebral disc was extracted and split into nuclear and annuals. The mesh was processed using meshing software (Hypermesh21; Altair, America).

**FIGURE 1:**
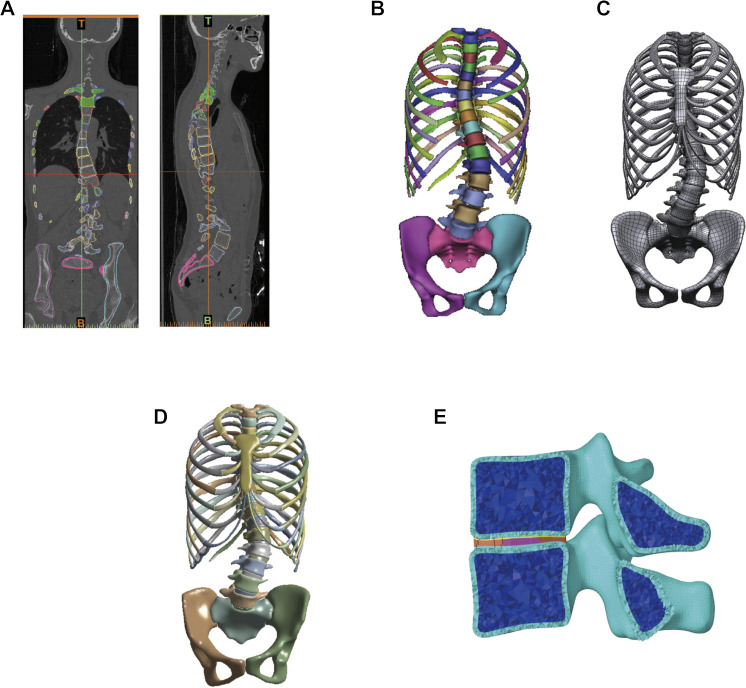
Establishment of the finite element model of patient with scoliosis. **(A)** Constructing the bone structure by using CT data of scoliosis patient. **(B)** Bone structure model. **(C)** Bone structure of reverse engineering into the geometric model and soft tissue establishment. **(D)** Global finite element model of scoliosis. **(E)** Finite element model of the vertebral segment.

The bone structure, which was built by tetrahedron elements, included the cortical and cancellous bone of vertebral bodies, pelvis, rib, sternum, sacrum, and costal cartilage. Ligaments, including the anterior longitudinal ligament, posterior longitudinal ligament, interspinous ligament, supraspinous ligament, ligamentum flavum, and intertransverse ligament, are simulated by spring elements using engineering simulation software (Workbench; Ansys, America). Intervertebral discs were built using hexahedral elements. Grid quality was fixed according to the Hypermesh default “Check item,” and all elements passed the inspection. The establishment process of the finite element model for scoliosis is shown in Figure 1.

### 2.2 Mechanical parameter

In this study, each component was regarded as an equilibrium elastic material, while the ligament structure of spinal functional units is stimulated by linear tension springs. The mechanical parameter of solid elements was taken from previously published data (Table 1). Table 2 shows the parameters of each ligament (Goel et al., 1993; Zhang et al., 2022). The flexibility of the intervertebral disc is hypothesized to affect the curve change (Clin et al., 2007), and further concern about osteoporosis is considered in this study. The transition mechanical parameter was calculated using the linear relation for personalized material classification.

**TABLE 1:** Material properties of each component.

Components	Young’s modulus E Mpa	Poisson’s ratio γ	References
Vertebral body			
Osteoporosis cortical bone	8040	0.3	Zizhou et al. (2018)
Transition cortical bone 1	9030	0.3	
Transition cortical bone 2	10020	0.3	
Transition cortical bone 3	11010	0.3	
Normal cortical bone	12000	0.3	Cheng et al. (2010)
Osteoporosis cancellous bone	34	0.2	Zizhou et al. (2018)
Transition cancellous bone 1	50.5	0.2	
Transition cancellous bone 2	67	0.2	
Transition cancellous bone 4	83.5	0.2	
Normal cancellous bone	100	0.2	Cheng et al. (2010)
Intervertebral disk			
Stiff annulus	2.1	0.45	Clin et al. (2010)
Transition annulus 1	3.15	0.45	
Normal annulus	4.2	0.45	Guo and Zhang (2022)
Transition annulus 2	6.3	0.45	
Flexible annulus	8.4	0.45	Clin et al. (2010)
Stiff nucleus	2	0.499	Clin et al. (2010)
Transition nucleus1	1.5	0.499	
Normal nucleus	1	0.499	Guo and Zhang (2022)
Transition nucleus2	0.75	0.499	
Flexible nucleus	0.5	0.499	Clin et al. (2010)
EX			
Rib	5000	0.1	Roberts and Chen (1970)
Sacrum	5000	0.2	Clin et al. (2007)
Sternum	5000	0.2	Clin et al. (2007)
Costal cartilage	480	0.1	Roberts and Chen (1970)
Pelvis	5000	0.2	Clin et al. (2007)

**TABLE 2:** Material properties of each ligament as spring.

Ligament	Young’s modulus E Mpa	Cross-sectional area mm2	Average length mm	Stiffness K N/mm
Anterior longitudinal ligament	7.8	22.4	20	8.74
Posterior longitudinal ligament	10	7	12	5.83
Interspinous ligaments	10	14.1	13	10.85
Supraspinous ligament	8	10.5	22	2.39
Intertransverse ligament	10	0.6	32	0.19
Ligamentum flavum	17	14.1	15	15.38

The intervertebral disc control group is composed of normal bone structure, while the bone control group is composed of normal intervertebral disc stiffness. To verify the difference between the most flexible and stiff spine, this study combines osteoporosis vertebral bodies with the most flexible intervertebral disc as, the most flexible spine. The stiffest spine is composed of normal vertebral bodies and stiff intervertebral discs.

### 2.3 Boundary conditions

Two experiments are used to test model verification, the bending film of the whole spine and the ROM (range of motion) of T1 to T4 are used to verify the biomechanical characteristics of the finite element models. This is done while taking into consideration the patient’s disc stiffness and Hounsfield unit of bone in CT. The Young’s modulus is determined from the density, which can be calculated in Hounsfield units. The Young’s modulus converted to vertebral cortical Hounsfield units is slightly higher than the cortical bone material parameters in the material parameters. For ease of calculation, normal cortical bone parameters are used for cortical bone and normal cancellous bone parameters are used for cancellous bone. However, unlike bone, it is not possible to obtain material parameters for discs using CT. During bending verification, three different mechanical properties of the disc (normal, flexible, and stiff) are considered. According to the distance measured in the bending film, displacement is applied to the T1 top surface, while the sacrum is fixed. Since the ROM of the spine model will change with spinal deformity, further regional segment biomechanical characteristics are approved by measuring the ROM from T1 to T4, which is less affected by scoliosis than other segments. Normal disc mechanical properties are used to established models. Pure moments in flexion/extension, lateral bending, and axial rotation were applied at 4 Nm in T1 top. The bottom of T4 were boned.

This study focuses on the effect of different mechanical parameters on the spinal orthosis. The loading conditions of the finite element model were based on the classical principle of the three-point system in the Cheneau brace. Force F1 was applied to the sixth, seventh and eighth ribs corresponding to the thoracic apex, while force F2 at the lumbar apex was applied to L2 and L3 to simulate the resultant force generated by the pad. Force F3 applied to the second, third and fourth ribs and the boned pelvis is the counterforce applied proximally and distally to F1 and F2 to satisfy the three-point system. Five groups of integrated three-dimensional boundary conditions are analyzed for the overall spinal orthosis, similar to the loading conditions of Karimi et al. (2019) (Table 3). The applied range of force varied between 0 N and 100 N in different direction.

**TABLE 3:** Various force configurations and magnitudes.

Condition	Force F1 at thoracic apex			Force F2 at lumbar apex			Force F3 at upper of thoracic curve			Boundary part
	(ML)	(AP)	(V)	(ML)	(AP)	(V)	(ML)	(AP)	(V)	
C1	50	0	0	25	0	0	25	0	0	Pelvic
C2	50	0	50	−25	0	25	−25	0	25	Pelvic
C3	50	0	100	−25	0	50	−25	0	50	Pelvic
C4	50	−25	50	−25	−25	25	−25	25	25	Pelvic
C5	50	−50	50	−25	−50	25	−25	50	25	Pelvic

The initial geometry of the patient was acquired by CT in the supine position, and gravity load can transform CT from a supine to a standing position for better understanding (especially at the biomechanical level). Gravitational forces are applied according to the loading method of Clin et al. (2010). All load conditions include applying a virtual gravity load followed by an orthopedic scheme load.

## 3 Results

**FIGURE 2:**
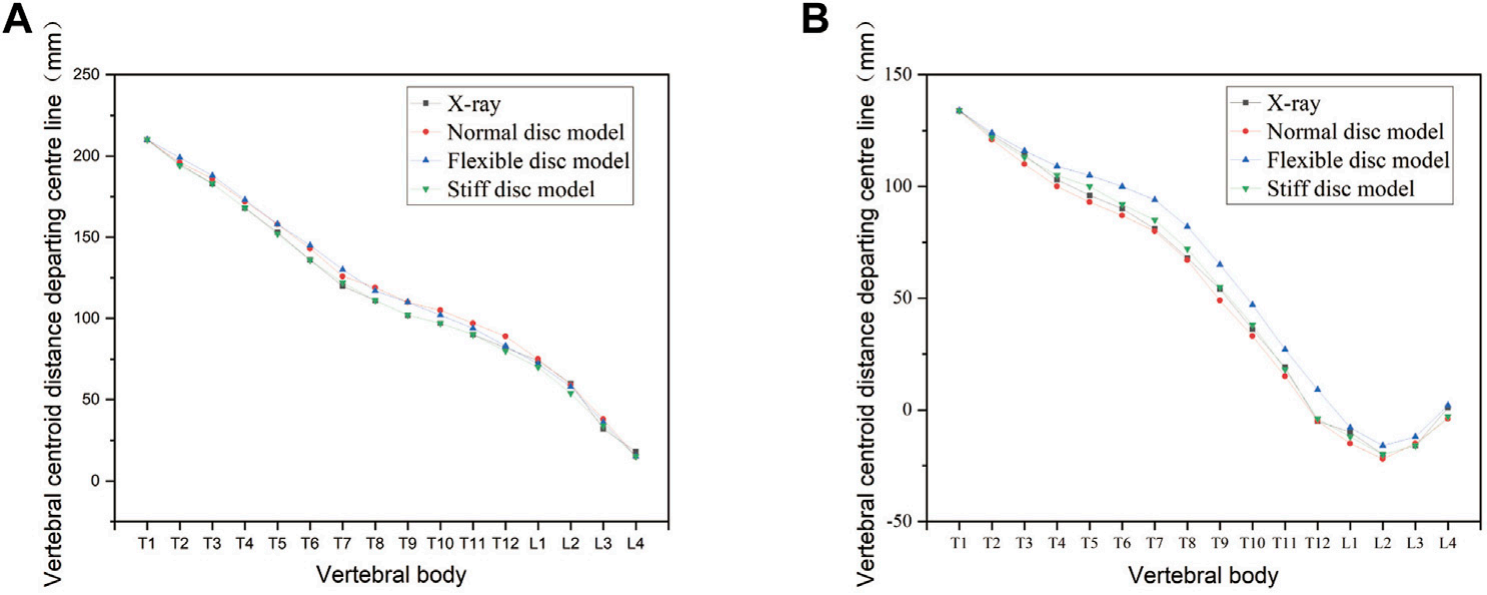
Changes in the bending curvature of the simulated spine with different spinal flexibility. **(A)** Left bending. **(B)** Right bending.

**FIGURE 3:**
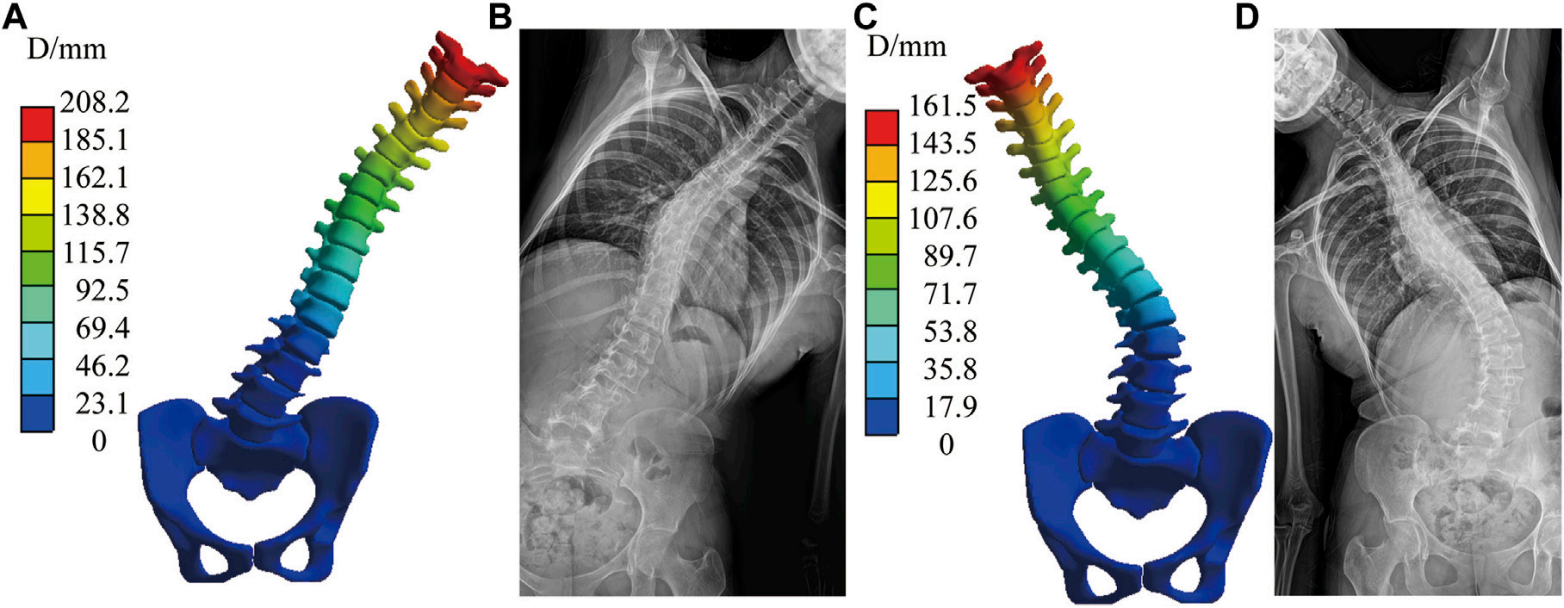
Simulated bending position of the stiff spine with bending film of X-ray. **(A)** Simulated left bending. **(B)** Left bending of X-ray. **(C)** Simulated right bending. **(D)** Right bending of X-ray.

**FIGURE 4:**
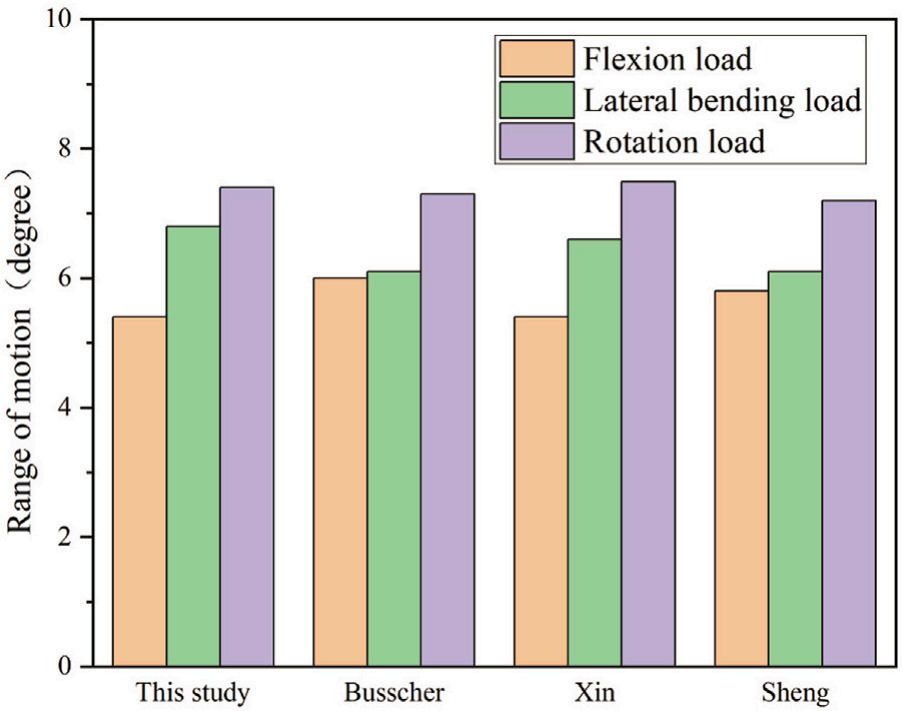
Range of motion among T1 to T4.

For the model validation section, the simulated bending position of the vertebral bodies is shown in Figure 2, and the stiff spine is the closest to the bending film of the three models (Figure 3). Figure 4 shows the ROM of T1 to T4, and the average stiffness are close to the comparison results (Busscher et al., 2010; Xin, 2016; Sheng, 2021). Therefore, the scoliosis models can be used for finite element analysis.

**FIGURE 5:**
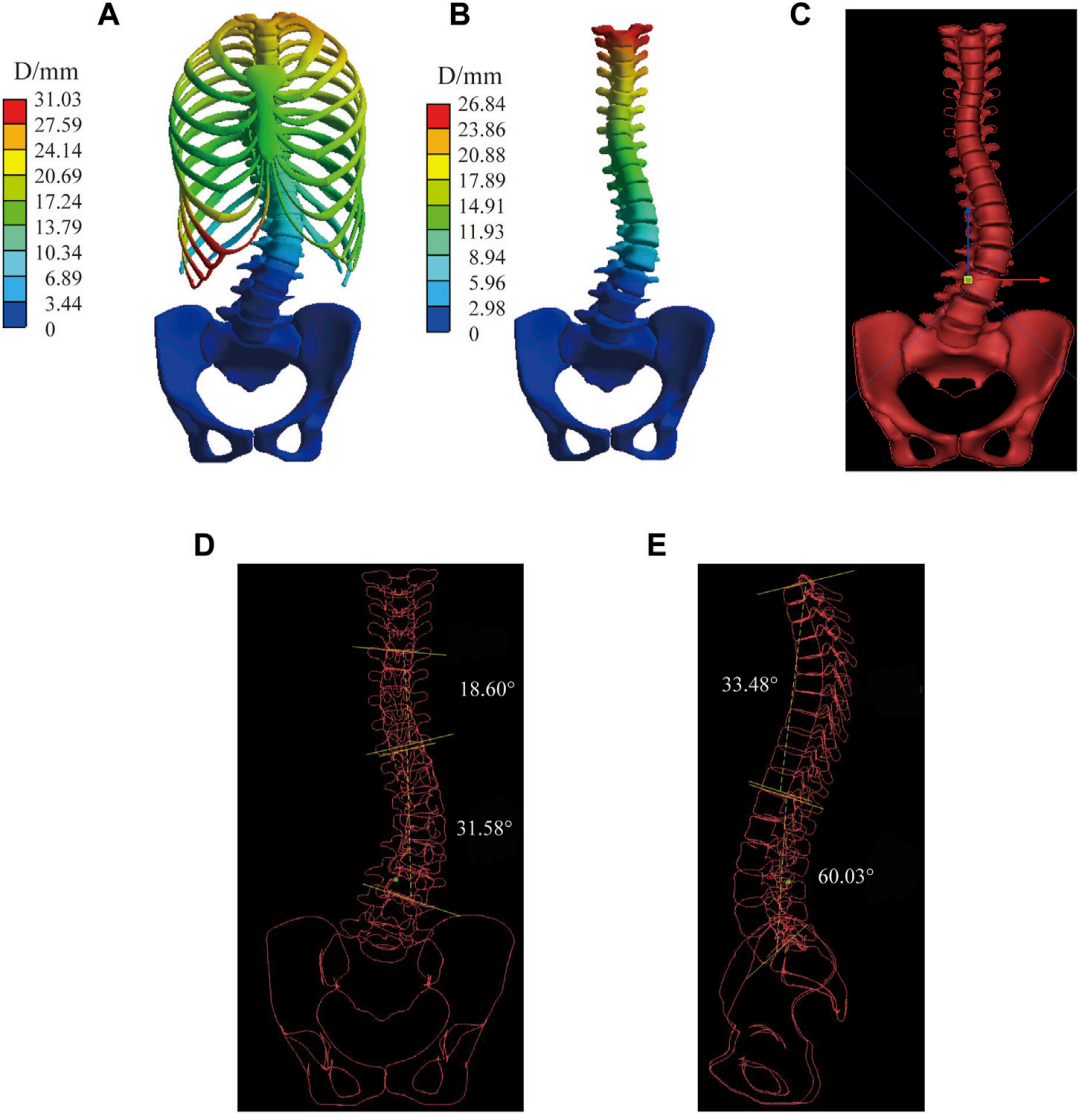
Measurement process for virtual X-ray in the most flexible spine in C1. **(A)** Displacement of the spine model. **(B)** Displacement of vertebral bodies. **(C)** Geometry model cast in virtual X-ray. **(D)** Measurement of the Cobb angle in the coronal plane. **(E)** Measurement of the Kyphosis and Lordosis angle in the sagittal plane.

The spinal index parameters under different force configurations and magnitudes are shown in Tables 4A, B. Cobb angle, thoracic Lordosis, and lumbar Kyphosis were measured after converting the “exporting.stl” file of the result to X-rays using the Virtual X-ray tool in Mimics (Materialise, Leuven), which used to allow engineers to create virtual X-rays of projects to find the optimal angle for 2D/3D registration (Figure 5). Additionally, axial rotation was measured by the Joint function in Workbench.

**TABLE 4:** Spinal parameters under different force configurations and magnitudes.

Panel A		Peak displacement mm	Average displacement mm	Cobb angle of thoracic curve degree	Cobb angle of lumbar curve degree	T1/T12 kyphosis degree	L1/L5 lordosis degree
C1	Intervertebral disc control group	17.56–18.18	6.01–7.91	19.6–23.3	32.7–35.8	35.5	60.1–60.2
	Bone control group	17.75–24.79	6.79–8.56	22.1–22.3	33.5	34.2–35.5	60.1
	Most flexible spine	26.84	9.98	18.6	31.6	33.5	60
C2	Intervertebral disc control group	25.7–38.36	9.55–14.69	14.7–19.3	25.2–28.6	34.3	59.1–59.4
	Bone control group	31.58–37.86	11.93–13.89	17–17.2	26.7–27.1	30.8–34.3	58.7–59
	Most flexible spine	45.69	16.94	14.6	22.9	30.7	58.4
C3	Intervertebral disc control group	40.54–58.09	15–22.08	16.1–19.3	19.4–25.3	31–31.4	57.2–57.5
	Bone control group	49.61–58.64	18.48–21.44	15.9–16.1	20.8–20.9	28.2–31.1	56.3–57.3
	Most flexible spine	67.6	25.22	15.6	19.1	27.9	56.1
C4	Intervertebral disc control group	37.23–49.16	11.71–16.32	16.2–18.2	25.1–28	30.9–31.1	59.7–60.1
	Bone control group	42.41–63.84	13.72–19.06	16.6–16.7	27.3–27.4	29.8–31.1	60–63.8
	Most flexible spine	81.22	25.01	16.1	25	26.7	65.9
C5	Intervertebral disc control group	54.1–61.94	15.6–18.61	16.1–19.8	23.6–28.1	29.5–30.9	60.9–61.6
	Bone control group	56.63–86.11	16.78–24.24	16.2	24.7–24.9	26.9–29.8	61.5–65.5
	Most flexible spine	104.2	29.84	16.1	23.6	26.5	67

Panel B		Rotation changes of T7	Rotation changes of L2	Peak stress MPa	T6-T7 KPa	T7-T8 KPa	L1-L2 KPa	L2-L3 KPa
C1	Intervertebral disc control group	0.22–0.29	0.87–1.04	37.3–39.81	66.7–103.7	146.6–304.9	251.8–371.3	89.8–213.8
	Bone control group	0.25–0.33	1.02–1.14	37.49–39.24	81.9–83.7	209.4–213.8	318.3–333.4	141.7–147.4
	Most flexible spine	0.37	1.15	36.06	65.2	143.2	277.8	95.5
C2	Intervertebral disc control group	0.34–0.46	0.7–1.37	43.06–45.85	29.8–120.9	111.1–312.5	361.3–564.1	210.9–338.9
	Bone control group	0.4–0.45	0.96–0.99	41.35–44.04	61.36–68.58	191.4–200.7	453.9–503	265.6–299.3
	Most flexible spine	0.49	1.49	42.78	30.6	128.1	397.6	237.6
C3	Intervertebral disc control group	0.6–1.34	1.15–2.66	48.32–52.02	59.4–235.4	229.2–569.3	796.2–1319	457.2–687.2
	Bone control group	0.78–0.84	1.61–1.65	47.33–50.66	124.1–169.2	371.3–387.3	1055.2–1145.4	561.2–598.9
	Most flexible spine	1.46	2.96	89.07	76.9	237.9	916.4	522
C4	Intervertebral disc control group	5.29–6.69	1.42–3.27	96.41–147.19	29.9–215	124.9–498.9	441.6–828.7	185–515.9
	Bone control group	5.93–8.97	2.12–2.97	108.92–123.99	44.7–227.8	212.4–389.6	509.2–846.3	284.7–405.2
	Most flexible spine	8.97	3.3	113.33	81.9	184.5	1450.1	213.5
C5	Intervertebral disc control group	6.66–10.7	2.6–3.81	109.42–209.92	69.5–379.1	153.5–735.1	777.2–1326.9	202.8–704.1
	Bone control group	10.31–15.1	3.1–4.16	115.53–142.34	107.9–331.6	306.7–669.5	965.4–1506.2	408.2–576.6
	Most flexible spine	16.69	4.28	184.06	126.6	303.6	1613.1	345.6

**FIGURE 6:**
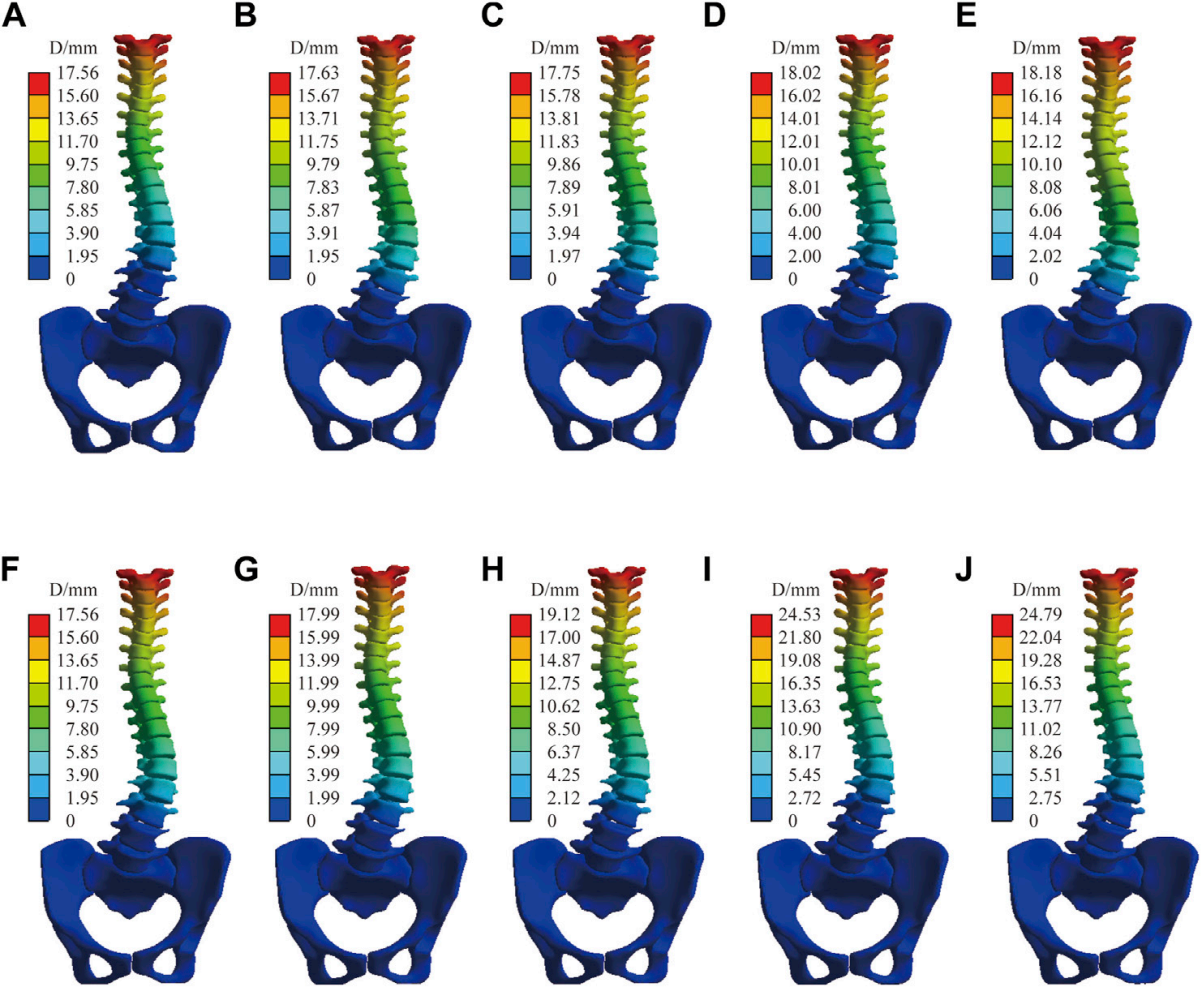
Spinal displacement distribution under C1. Bone control group: **(A)** Stiff intervertebral disk. **(B)** Transition intervertebral disk 1. **(C)** Normal intervertebral disk. **(D)** Transition intervertebral disk 2. **(E)** Flexible intervertebral disk. Intervertebral disc control group: **(F)** Osteoporosis vertebral body. **(G)** Transition vertebral body 1. **(H)** Transition vertebral body 2. **(I)** Transition vertebral body 3. **(J)** Norma vertebral body.

Figure 6 shows the displacement distribution of the spine under C1, the effect of different mechanical parameters on the displacement distribution of the spine is not significant, and there is only a certain difference above the displacement peak. This result is applicable to the other four sets of boundary conditions, the common characteristics are shown in the transition mechanical parameter group, only displacement scale difference. The stiffer the material parameter, the lower the peak displacement of the spine. Combined with Table 4, the Cobb angle decreases with an increase of the modulus of the intervertebral disc, and the bone has little influence on the Cobb angle (which varies within 1°). Lordotic and Kyphotic angles are less affected by the intervertebral disc and basically vary within 1° but are more affected by bone, except Lordotic angels of interverbal disc control group under C5 has difference of 1.4°. The Kyphotic angle increases with the increase of the bone modulus. Under C4 and C5, the Lordotic angle increases with the decrease of the bone modulus, while there was no significant difference without sagittal force.

**FIGURE 7:**
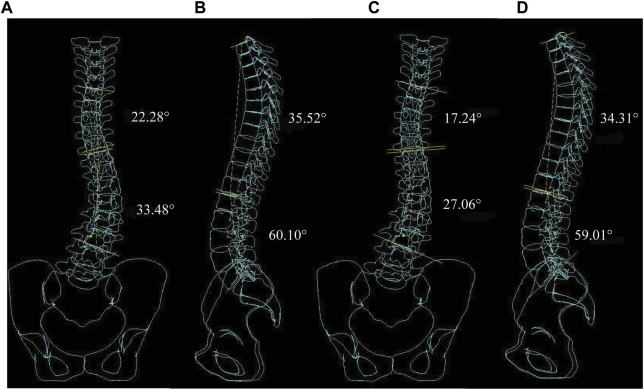
Virtual X-ray of normal spinal. **(A)** Coronal plane Cobb angle measurement in C1. **(B)** Sagittal plane Kyphosis and Lordosis angle measurement in C1. **(C)** Coronal plane Cobb angle measurement in C2. **(D)** Sagittal plane Kyphosis and Lordosis angle measurement in C2.

Figure 7 shows the normal spinal coronal virtual X-ray under C2 and C1, the thoracic and lumbar Cobb angle of C1 is significantly lager than C2 while the Lordosis and Kyphosis angle is slightly lager than C2. IBC were shown as a reducutin ratio of Cobb angle with brace. Under all material assembly protocols, The thoracic IBC of C1 ranges from 10.3% to 28.4%, while C2 ranges from 25.8% to 43.5%. The lumbar IBC of C1 and C2 are 10.5%–21.3% and 28.5%–42.8%, respectively. A higher correction indicates that the spine is more likely to be corrected with a brace. C2 with transverse force can effectively reduce the Cobb angle of the thoracic and lumbar curve, Lordosis and Kyphosis angle compared with C1.

**FIGURE 8:**
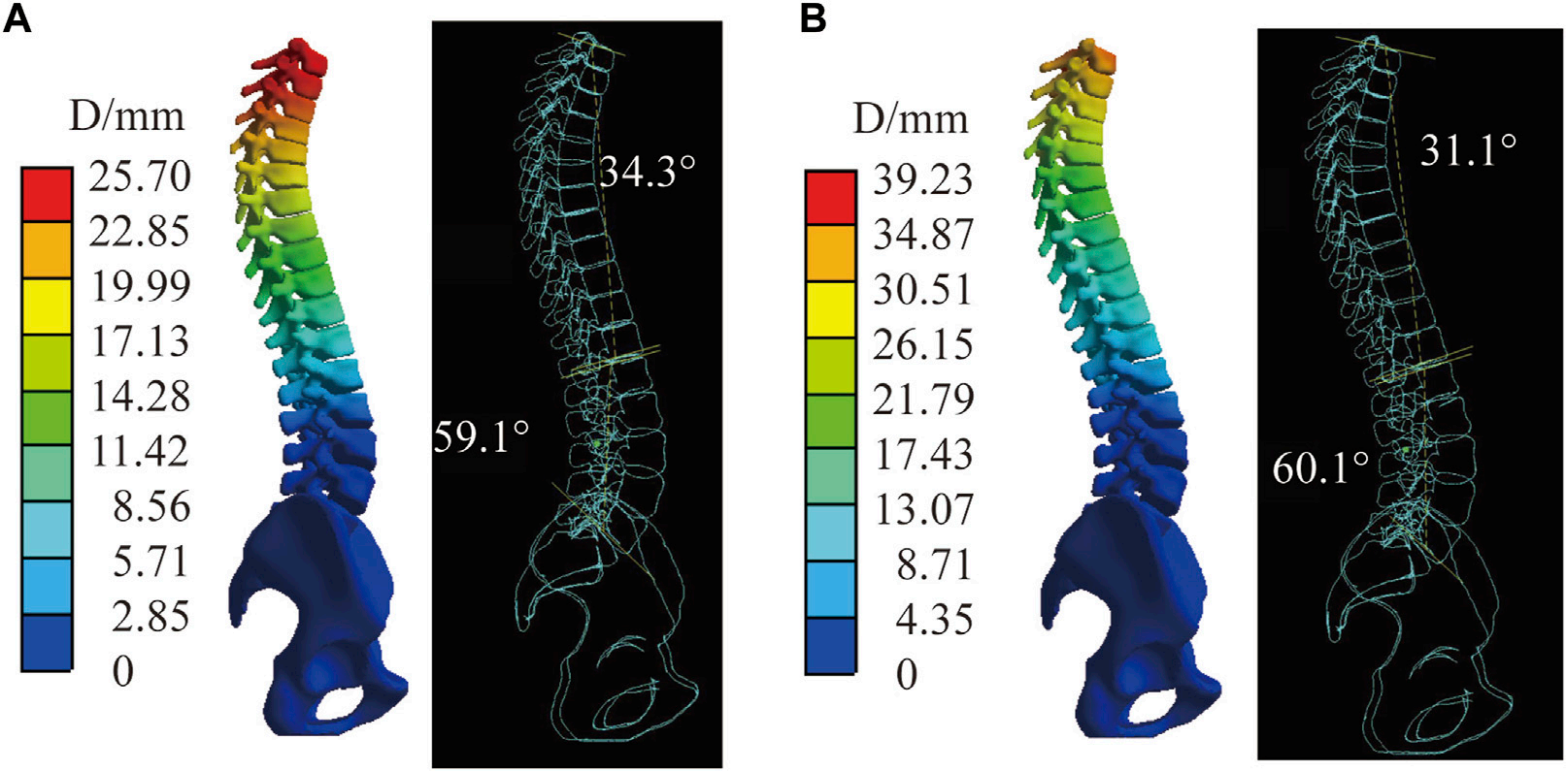
The stiffest spinal in C2 and C4. **(A)** Displacement distribution and virtual X-ray of sagittal plane in C2. **(B)** Displacement distribution and virtual X-ray of sagittal plane in C4.

Figure 8 shows the stiffest spinal displacement distribution under C2 and C4, forces in the sagittal direction has a large effect on the displacement distribution. Combined with the Table 4, the thoracic and lumbar IBC of C4 is 30%–38.1% and 30%–37.5% under all material assembly protocols, respectively. Compared with C2, the increase of sagittal force in C4 will also reduce its Lordosis angle but increase its Kyphosis angle. Negatively impacts the correction of the Cobb angle in some flexible mechanical parameter. Increasing force on the sagittal and transverse planes like C3 and C5 will have a positive influence on the displacement of the spine, but not all the spinal index parameters according to Table 4.

The maximum thoracic Cobb angle that varied due to material parameters are 4.7°, 4.7°, 3.7°, 2.1°, and 3.7° respectively under five force configurations, while for lumbar Cobb angle variation material parameters are 4.2°, 5.7°, 6.2°, 3°, and 4.5°. The maximum thoracic and lumbar Cobb angle difference converted to thoracic and lumbar in-brace correction difference is 18% and 15.5%. In intervertebral disc control group, both thoracic and lumbar Cobb angle variation are higher than that in bone control group. The Kyphosis and Lordosis angle variation are inverse to Cobb angle. No obvious pattern is found in the change of peak displacement of between both group. The most flexible group (osteoporosis bone group comebined with flexible disk group) has the largest displacement, rotation changes, while has the smallest thoracic and lumbar angle under all boundary conditions. The Kyphosis and Lordosis angle is minimum under C1, C2, and C3 in most flexible group. While the Lordosis angle become maximum with the inclusion of sagittal plane forces under C4 and C5.

**FIGURE 9:**
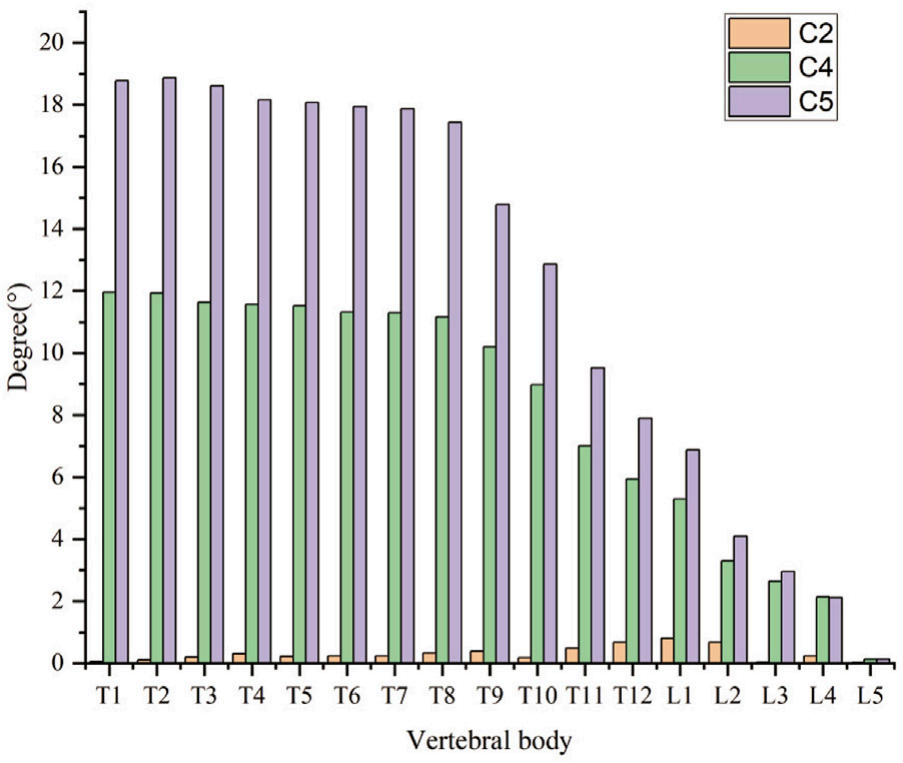
The axis rotation of the vertebral body of the most flexible spine in C2, C4, and C5.

The Workbench Probe function was used to obtain the changes in the axial rotation of the vertebral body. The sagittal force had the greatest effect on rotation compared with the forces in the coronal and axial directions, as is shown in C2 and C4. The axial rotation of the vertebral body has a specific relationship with the intervertebral disc and bone, and the rotation angle decreases with the elastic model of the bone and the intervertebral disc. In the sagittal plane, the correction of rotation degree is most apparent in C4 and C5 under the loading condition, and the change of rotation degree of the thoracic spine is greater than that of the lumbar spine. The most flexible spine axis rotation of the vertebral body is measured at C2, C4, and C5 (Figure 9). Intervertebral disc stiffness has the greatest influence on Cobb angle and decreases with increased intervertebral disc modulus. Bone stiffness has the largest change value in Kyphosis and increases with the increase of bone elastic modulus. The bone and intervertebral disc both significantly influence the axial rotation of the vertebral body.

**FIGURE 10:**
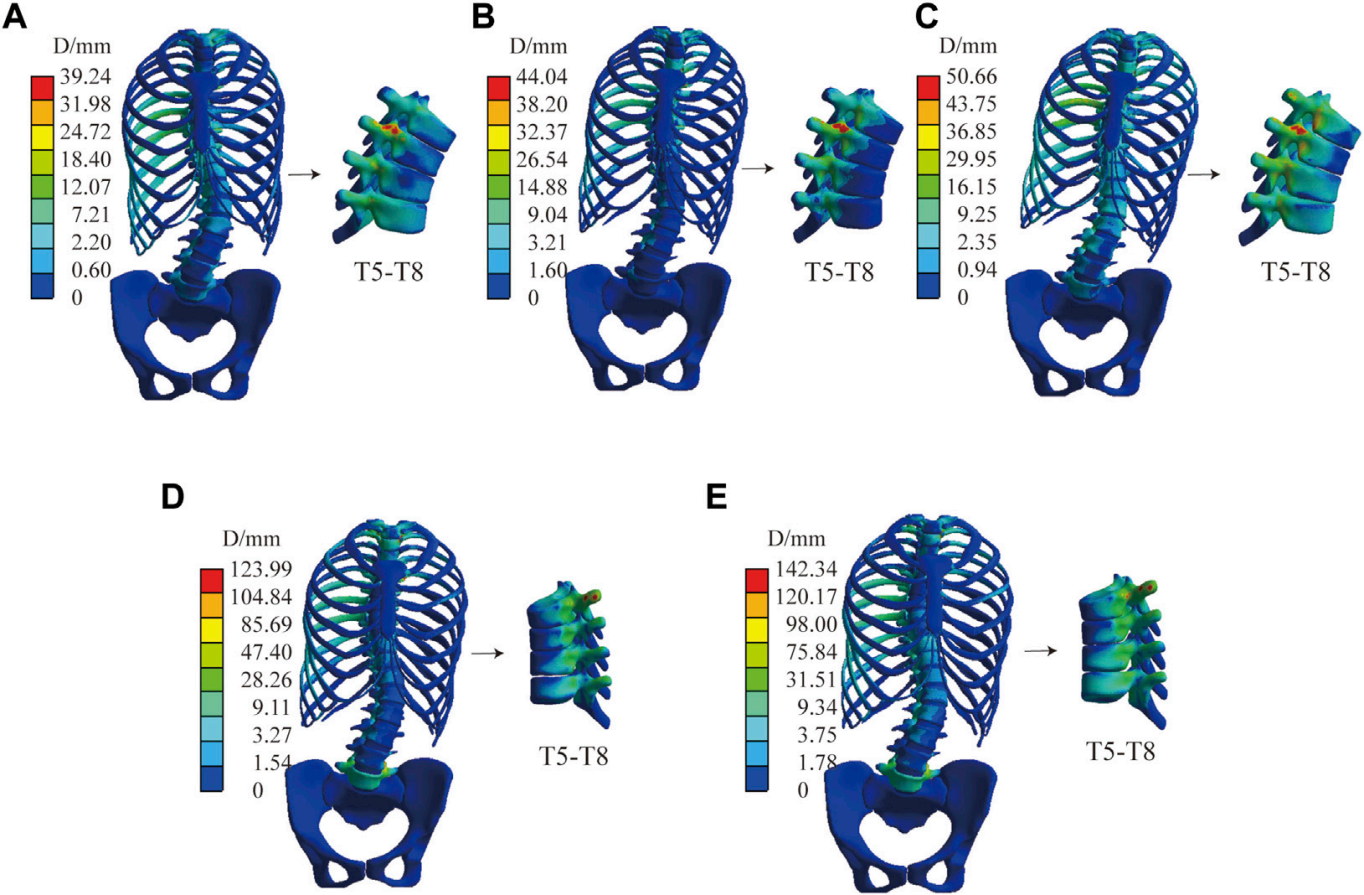
The stress distribution of normal spine. **(A)** Stress distribution in C1. **(B)** Stress distribution in C2. **(C)** Stress distribution in C3. **(D)** Stress distribution in C4. **(E)** Stress distribution in C5.

Figure 10 shows that the stress distribution under five force configurations in normal spine. The stress of cortical bone was greater than that of cancellous bone. The peak stress occurred at the junction of T6 and sixth rib on the convex side of cruve under C1, C2, and C3. While under C4 and C5, it occurred at the T5 and fifth rib on the concave side of cruve. Stress mainly distribute in the ribs and the posterior border of vertebral body. In addition, a decrease in the elastic modulus of the intervertebral disc leads to an increase in the overall model stress; however, the opposite occurs for the bone (Table 4).

This study assessed the intervertebral disc between T6 and T7, T7 and T8, L1 and L2, and L2 and L3. Under the condition that only minor transverse and longitudinal loads are considered, the maximum stress of the stiffness of the intervertebral disc is affected the most, and the peak stress of the most flexible intervertebral disc is at a minimum. Moreover, the elastic modulus of bone has a limited effect on stress. After the orthopedic load in the direction of the Z-axis was increased for C3, the stress peak caused by bone changes had a specific change. When the sagittal plane loading was added to C4 and C5, the stress peak generated by the elastic modulus of bone changed significantly (Table 4).

**FIGURE 11:**
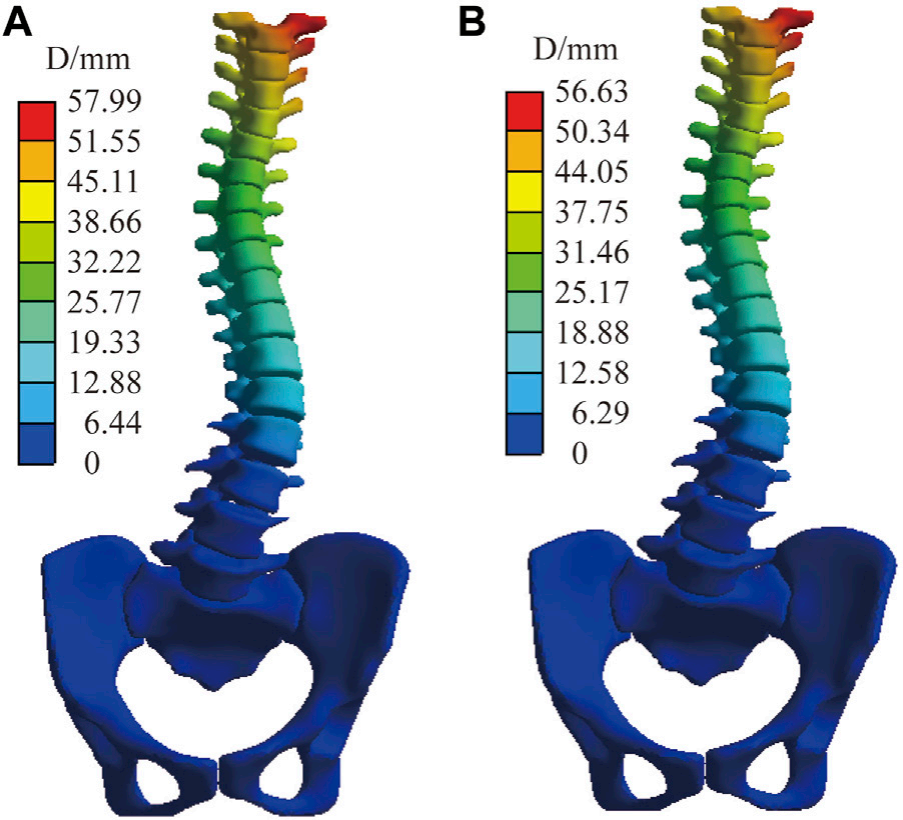
The displacement distribution of normal spine under C5. **(A)** Finite element model with ligaments. **(B)** Finite element model without ligaments.

In addition to the above models, we also compared the displacement comparison between the model without ligaments and those with ligaments using the same material properties. Figure 11 shows, under C5, the displacement distribution of the two models is similar, with a peak displacement difference of 1.3 mm. The rest of the models vary less than the boundary condition under C5.

## 4 Discussion

Personalized customization braces for scoliosis are a major topic of interest. Park et al. (2018) designed a robotic spine exoskeleton to treat spine deformity. Dong and Wu (2018) innovated a brace with a magnetic force device to correct scoliosis deformity. They demonstrated a traditional brace, improved with topology optimization, that promised immediate correction. This brace was lighter, thinner, and covered less surface area on trunk. In future, more research will be conducted on the intersection of new technologies for better scoliosis treatment.

Thus, applied force instead of the loading displacement on bone structure in this study can enable better observation of responses to mechanical loading according to different spine stiffness. Few studies based on the FEM mechanical properties sensitivity analysis are used to improve the accuracy of the finite element model. Petit et al. (2004) evaluated spine flexibility as intervertebral disc stiffness. In this study, intervertebral disc stiffness influences a 3D plane, and the bone substance is studied. This study aims to improve the effectiveness of the scoliosis model but also to provide baseline data for simplifying the scoliosis model in the future.

In the same mechanical property analysis, the result confirms the following points. The vertical upward force component can improve the scoliotic curve as was shown in C1 and C2. Increased force does not yield better outcomes in brace treatment. Instead, a sagittal pair-of-force system can effectively correct axial rotation and influence the thoracic more than the lumbar, while sagittal force significantly impacts the Lordosis and Kyphosis angle. These findings are also supported by Karimi et al. (2020) and the Chêneau-type brace design principle (Rigo and Jelacic, 2017). A different view is based on the different stiffness of the components. Both the vertebral body and intervertebral disc influence the displacement of the brace treatment. In this work, the displacement increases with a decrease in modulus. The intervertebral disc mechanical parameter in the coronal plane is much higher compared with the sagittal plane, while vertebral stiffness is the opposite. Clin et al. (2010) found similar results in intervertebral disc stiffness, where the axial rotation of the vertebral body was affected by both stiffnesses.

Changes in material parameters cause scale changes in brace treatment for the same boundary conditions, no structural changes are found in this study. As the overall stiffness of the spine increases, the smaller the deformation of the spine for the same force. In the coronal plane, the Cobb angle is more influenced by changes in the material parameters of the disc and less influenced by the material parameters of the bony structure. In the sagittal plane, the anterior and posterior convex angles are more influenced by the bone. The specific causes are related to the structure of the vertebral segments. In the cross-section, the material parameters of both bone and disc have a significant effect on the axial rotation of the vertebral body. At the same time, the orthopedic effect of the brace is further enhanced when osteoporosis is combined with a lower disc modulus of elasticity. Although the orthopedic effect for some flexible cases in the coronal plane curve is excellent, it should be noted that the sagittal plane spinal curve is also significantly altered, which may cause flat back problems.

For stress distribution, according to the Hueter-Volkmann principle (compressive stresses slow growth, whereas tensile stresses speed up growth), the distribution of the disc vertebral growth plate is a significant indication for Adolescent Idiopathic Scoliosis (AIS) (Kamal and Rouhi, 2020). Because the patient was an adult, this study did not focus too much on the stress distribution of the growth plates. The stress distribution of the intervertebral disc, with changes in mechanical parameters, affects the stress distribution of the intervertebral disc. Thus, in brace design, it is necessary to pay attention to the bending moment balance on the growth plate and mechanical property validation.

The effect of ligaments on scoliosis is negligible; the peak displacement in normal C5 differs by only 1.3 mm (with or without ligaments). In the computer simulation, the primary reason is that the ligament is set as a linear material and is in a state without preloading force. Additionally, during the simulation process, the corresponding surface displacement at both ends of the ligament is small; therefore, the tension between the two points is almost negligible. However, this may be inconsistent with reality. In future studies, we will consider setting a standard length of the ligament and a preloading force based on the standard length difference.

This study has many limitations. The non-linear material parameters (from muscles and ligaments) are not considered. To address this, we will constantly improve our standard finite element model. This model is only effective for the single patient case study. To deal with larger workloads in constructing finite element models (and ethical issues), we will use EOS Imagine and artificial intelligence in future studies. In subsequent studies, more reasonable and detailed boundary conditions to analyze different types of scoliosis as well as brace assembly verification will be considered, and part of the manual angle measurement will be based on a virtual X-ray. In follow-up studies, we will also assess the corresponding automatic measurement software to reduce manual measurement error.

## 5 Conclusion

This study focused on the different biomechanical properties of spine stiffness and considered the modulus of the patient’s disc and bone as the key to increasing accuracy in the personalized finite element model. In the coronal plane, the Cobb angle is more influenced by changes in the material parameters of the disc and less influenced by the material parameters of the bonal structure. In the sagittal plane, the anterior and posterior convex angles are more influenced by the bone. In the cross-section, the material parameters of both bone and disc have a significant effect on the axial rotation of the vertebral body. Future studies will focus on the biomechanical analysis of many different types of scoliosis patients by FEM, providing the corresponding data and simplification method for the subsequent automatic generation of scoliosis models.

## Data Availability

The original contributions presented in the study are included in the article/supplementary material, further inquiries can be directed to the corresponding author.
